# The Soil and the Mind

**Published:** 1870-04

**Authors:** 


### THE SOIL AND THE MIND.

CULTURE improves both, but both, like the body, must have nourishment. Food feeds the body, thought feeds the brain, and fertilizers feed the soil.

The better the food, the better the thought; the better the fertilizers, the more vigorous does the body become, the greater the activity of the mind, and the higher productiveness is there in the soil. By the better living of the last half century, ten years have been added to the average of human existence in civilized lands. By the greater advantages of education offered during the same period, investigations of natural truth and science have immeasurably surpassed in their additions to human knowledge any dozen previous half centuries, and coming down to lesser things, English lands which grew ten bushels of wheat a few years ago, now grow over thirty on an average, simply because the soil is better fed with manures. Hence we may reasonably expect that, with a wiser feeding of the body, providing it with better things, with more richly flavored fruits, with sounder, healthier, and more juicy meats, and more highly cultivated grains which make bread, we may still add to the length of life's duration, and a man may be younger at a hundred than he is now at three-score. The good things of this life were certainly made to be enjoyed, rationally, temperately, thankfully, and lovingly towards Him who supplies them all.

